# The Nutritional Condition of Individuals With Alcohol Use Disorder Following Bariatric Surgery: A Systematic Review

**DOI:** 10.7759/cureus.96008

**Published:** 2025-11-03

**Authors:** Farhana Nazmin, Parth U Modi, Sabiha Akter, Fatima Chowdhury, MD Saidur Rahman, Rishika Gadde

**Affiliations:** 1 Psychiatry, Bronx Community High School (BCHS), Bronx, USA; 2 Medicine, Medical College Baroda, Vadodara, IND; 3 Psychiatry, Interfaith Medical Center, Brooklyn, USA; 4 Psychiatry, Nassau University Medical Center, East Meadow, USA; 5 Psychiatry, Middle Tennessee Mental Health Institute, Nashville, USA; 6 Internal Medicine, Jawaharlal Nehru Medical College, Belgaum, IND

**Keywords:** alcohol use, bariatric surgery, gastric bypass, nutritional condition, nutritional condition bariatric surgery, systematic review

## Abstract

There has been a surge in increased risk of alcohol use disorder (AUD) in patients with bariatric surgery. The aim of this review is to find the post-surgical outcomes by determining the extent to which AUD affects the nutritional health and examine the correlation of alcohol consumption, nutritional status, and the sequelae of poor nutritional status. A thorough analysis of the Preferred Reporting Items for Systematic Reviews and Meta-Analyses (PRISMA) guidelines analogy was conducted by gathering articles published from 2013 to 2024, including adult metabolic bariatric surgery patients with a history of alcohol intake from databases such as PubMed, Cochrane, and Science.gov, and Google Scholar was used as a supplementary research gate. Twelve finalized studies met the inclusion criteria. Results point to a higher incidence of AUD after bariatric surgery, especially after sleeve gastrectomy or Roux-en-YRYGB, or gastric bypass surgery. Following surgery, there was substantial evidence of elevated alcohol sensitivity and AUD symptoms, which could have negative health repercussions. These findings emphasize the significance of thorough preoperative evaluations and customized bariatric therapies for AUD patients undergoing surgery.

## Introduction and background

There is currently a severe obesity epidemic in a growing number of high- and middle-income nations. Severe obesity is predicted to double in high-income populations from 10% to 20% between 2020 and 2035, presenting a serious risk to healthcare systems [1]. Obesity is defined as a body mass index of 30 kg/m^2^ or higher [2]. Weight-loss surgery became common worldwide in 1991 after a decision made at a National Institute of Health (NIH) meeting [3]. Before that, a surgery called jejuno-ileal bypass had risks at a later stage, like diarrhea and problems with electrolytes [3,4]. 

After checking the results from surgeries in 1991, experts agreed that Roux-en-Y gastric bypass (RYGB) and vertical banded gastroplasty (VBG) were safe and the best options for patients with a BMI over 40 who had serious health issues because of their obesity [3]. This agreement brought in a new standard of doing things, which filled a hole and proved that weight-loss surgery was its own surgical field [4]. It became more commonly done, and since then, there has been more and more proof that weight-loss surgery works. Additionally, the two most frequent bariatric operations, sleeve gastrectomy (SG) and RYGB, increase the likelihood of alcohol-related disorders after surgery compared to pre-surgery and other bariatric procedures. The Alcohol Use Disorders Test (AUDIT) showed that patients receiving RYGB had twice the risk of incident alcohol use disorder (AUD) symptoms compared to those undergoing laparoscopic adjustable gastric banding (LAGB). Additionally, one-fifth of participants reported incident AUD symptoms within five years after RYGB. Pre-surgery regular (drinking ≥ twice per week) or problematic alcohol usage, male sex, younger age, and the kind of operation are some risk factors for post-surgical alcohol problems [5-7]. 

A pattern of alcohol use that endures despite detrimental effects on one's health and well-being, which characterizes AUD. AUD is more likely to occur in people who have had at least some form of bariatric surgery, according to studies [8,9]. According to Krzizek et al., postoperative micronutrient deficiency is a well-known side effect of metabolic bariatric surgery (MBS) that is likely caused by changed eating habits and the operation's malabsorptive component. Most studies focus on evaluating a small number of micronutrients in smaller cohorts, such as vitamin D, vitamin B12, and ferritin [10]. The cause of AUD after bariatric surgery is multifaceted and includes morphological, metabolic, and neurohumoral alterations linked with post-surgical anatomy [11]. 

Rationale

This study looks at the nutrition of people with alcohol problems after they undergo weight-loss surgery. After this surgery, the body handles nutrients differently, which can make existing nutrition problems worse, especially for those with alcohol issues. Also, alcohol might get into the bloodstream faster after surgery, causing issues and messing with how the body absorbs nutrients. It's super important to know how alcohol problems and weight-loss surgery affect each other so doctors can plan better care after surgery and avoid problems, helping to improve the long-term health of these patients. 

Objectives

The purposes of conducting the research are to: (i) To examine the previous research on the impact of AUD on nutritional deficiency and metabolic disorders in post MBS patients; (ii) To review and explore the literature on the facts concerning the nutritional health of individual with AUD post-bariatric surgery; (iii) To probe the articles on the association between alcohol use and the postoperative nutritional effects specifically to nutrition absorption and metabolism; (iv) To identify and state the risk factors leading to poor nutritional health in patients with AUD post MBS as reported in the literature; (v) To explore the inconsistencies and gaps in the evidence and generate rationales for further studies in the scope of nutrition emphasizing on post-bariatric patients with AUD. 

## Review

Materials and methods

We conducted an electronic search across multiple databases in PubMed, Cochrane, Science.gov and Google Scholar as a supplementary research gate, from 2013-2024, to collect data on AUD post-bariatric surgery. Keywords such as "alcohol consumption," "nutritional status," "metabolic profile," "bariatric surgery," "metabolic surgery," and "Roux-en-Y gastric bypass" were used in combination with Boolean operators "AND" and "OR" to refine the search. Additionally, manual searches were performed to support our findings, including an exploration of databases such as the DEA website. Further details on the databases and search strategies owned are outlined in Table 1. 

**Table 1:** Databases, Search Strategy, and Tally of Publications Selected MeSH: Medical Subject Headings

Database	Search Strategy	Number of Publications Reviewed
PubMed	Alcohol intake OR alcohol consumption OR "alcohol drinking/adverse effects" [Majr] OR "alcohol drinking/metabolism" [Majr] OR "alcohol drinking/pathology" [Majr] OR "alcohol drinking/physiopathology" [Majr] AND "Bariatric Surgery/education" [Majr] AND "nutritional profile OR metabolic profile OR alcohol physiology OR alcohol metabolism.	60
Cochrane	How does drinking alcohol affect the nutritional and metabolic health of people who have had weight loss surgery, such as gastric bypass or gastric sleeve?	04
Science.gov	Bariatric Surgery AND Alcohol Use	20
Google Scholar	Following bariatric surgery, AND person's alcohol use and nutritional status are closely related.	4550

Eligibility criteria

Inclusion and Exclusion Criteria

Studies on alcohol and AUD that involved adults (≥18 years) who had received MBS were selected. Studies that documented alcohol consumption, AUD prevalence, and post-operative outcomes, which were published in English in peer-reviewed journals with impact factors between 2013 and 2024, were included. Exclusion criteria included studies involving children, animals, in vitro studies, editorials, systematic and narrative reviews, commentaries, letters, reviews, conference abstracts containing no primary data, or studies presented as abstracts.

Selection process

**Figure 1:**
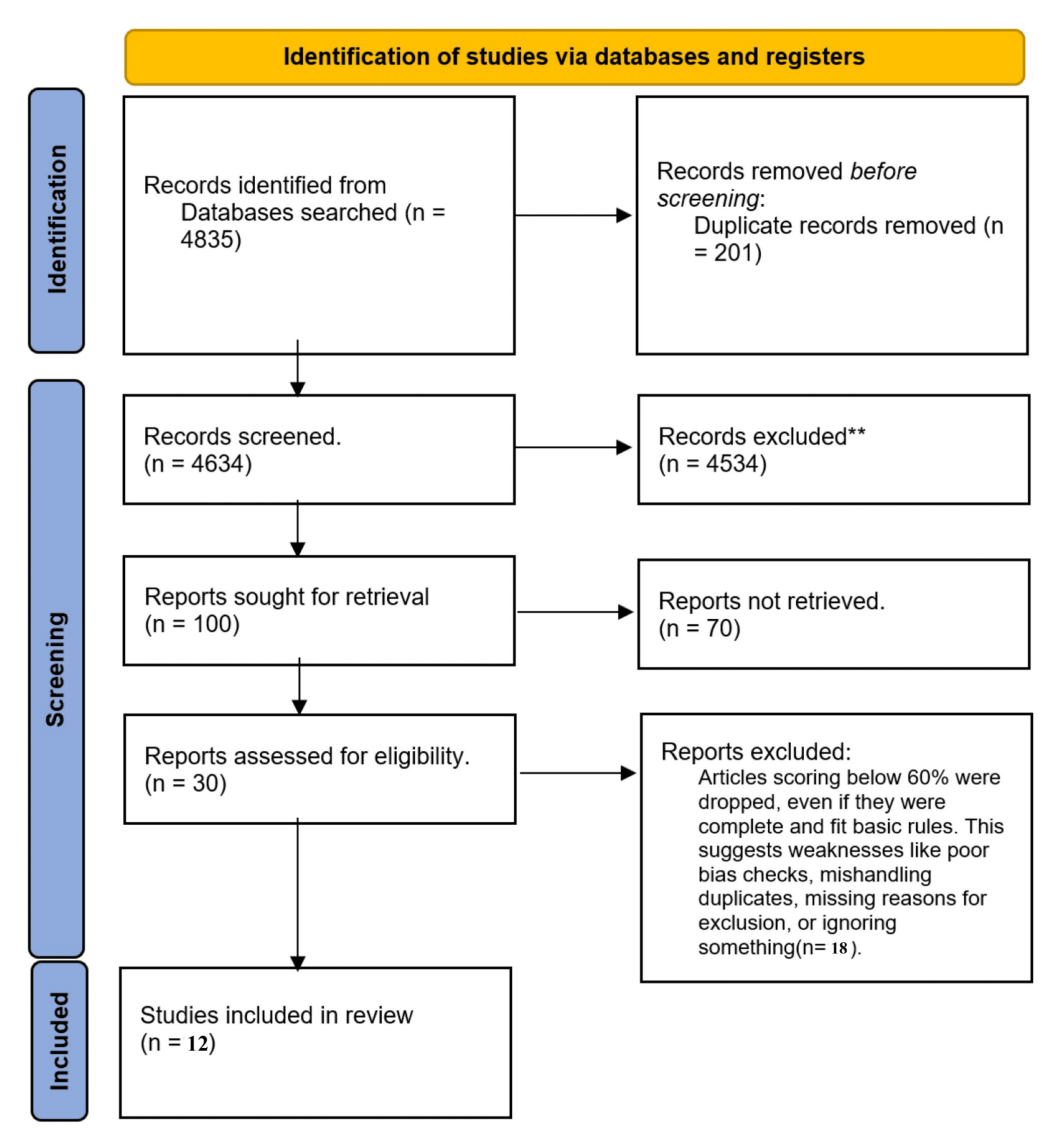
PRISMA Flowchart PRISMA: Preferred Reporting Items for Systematic Reviews and Meta-Analyses

Each study underwent a detailed manual search evaluation, giving a focus on data meeting our pre-set determined inclusion criteria. Duplicate articles, amounting to 201, were excluded at the initial stage (Figure 1). Of the initially available 4634 articles, an additional 4534 articles were excluded for not fulfilling our criteria. We were left with 100 articles to review for the study, but full-text access was unavailable for seventy of them. Twelve of these articles, fulfilling the acceptable methodological quality using a validated tool, satisfied our inclusion criteria and fulfilled the requirements of the study.

All the studies included indicate that after MBS the risk of developing AUD is high. Cohort studies by Bramming et al. also indicate that 'surgery' population have higher 'AUD' cases compared with controls [12]. Obesity treated with different weight control approaches, RYGB was linked with increased odds of AUD-concerned hospitalizations versus SG [13]. Siikaluoma et al. reported the prevalence of alcohol overconsumption measured with Phosphatidylethanol two years after MBS was 8.3% and it was correlated with male sex, older age, and hypertension [14]. Subjective sensitivity to alcohol increased after surgery for those who met objective criteria for problematic alcohol use and had significant psychiatric comorbidities, whereas frequency and quantity of drinking showed less change over time [15]. Patients may turn to alcohol as a coping mechanism due to the emotional toll of being disappointed with their weight or surgery results [16]. About 17 months following RYGB surgery, some patients began drinking, and by 37 months, they satisfied the requirements for AUD, which was severe enough to necessitate admission to an intense addiction treatment center at 65 months [17]. Compared to nonsurgical counterparts, patients who had RYGB lost significantly more weight, and the majority of this weight loss was maintained over time [18]. After surgery, the experience of drinking is more rewarding than it was before because the increased blood alcohol concentrations are linked to even higher degrees of reinforcement [19]. Compared to patients who have had restriction surgery as a treatment for obesity, patients who have had MBS as a treatment for obesity are more likely to have been hospitalized with an alcohol-related diagnosis [20]. Following WLS, patients who have laparoscopic Roux-en-Y gastric bypass surgery may be more likely to drink alcohol [21]. A higher sense of intoxication and earlier and enhanced blood alcohol concentration peaks are the results of RYGB's increased rate of ingested alcohol transport into the blood [22]. The development of AUDs following bariatric surgery is influenced by a number of biologically related mechanisms as well as psychological and social factors (Table 2) [23]. 

**Table 2:** Characteristics of the Included Studies AUD: Alcohol use disorder; RYGB: Roux-en-Y gastric bypass; MBS: metabolic bariatric surgery

Study/year	Location	Study design	Sample size	Type of surgery	Outcome
Bramming et al., 2021 [12]	Denmark	Cohort study	13430	MBS and AUD	Increase AUD and RYGB-related risk reported
Mahmud et al., 2023 [13]	USA	Cohort study	1854	MBS	Prolonged hospitalization and increased mortality reported
Siikalouma et al., 2022 [14]	USA	Cohort study	410	MBS	Increased risk factors and post-surgery alcohol overdose reported
Smith et al., 2018 [15]	USA	Observational study	26	MBS	Post surgery, alcohol consumption reported
Reaves et al., 2019 [16]	USA	Retrospective study	14	MBS	Post surgery, alcohol-related problem reported
Cuellar-Barboza et al., 2015 [17]	USA	Retrospective study	800	Gastric bypass and alcohol use	Altered patterns
Maciejewski et al., 2016 [18]	South Korea	Observational study	350	Gastric bypass and alcohol use	Long-term weight loss and alcohol consumption
Engel et al.,2022 [19]	USA	Cohort study	34	MBS	Changes in pharmacokinetics
Ostund et al., 2013 [20]	Sweden	Retrospective study	11115	Gastric bypass Surgery and Alcohol Use	Increased risk of alcohol use after surgery
Conason et al., 2013 [21]	USA	Prospective study	155	MBS	Post-surgery alcohol use
Pepino et al., 2015 [22]	USA	Longitudinal study	22	MBS	Changes in alcohol pharmacokinetics
Tvedt et al., 2023 [23]	USA	Interview based study	10	Gastric bypass Surgery and Alcohol Use	Post-surgery patient experiences with alcohol use

Discussion

Research, both clinical and preclinical, has explored the association between metabolic or bariatric surgery and the risk of alcohol consumption and the development of AUD or alcohol addiction. Maciejewski et al. [18] noted that individuals who have bariatric procedures may face a higher risk of developing AUD after surgery. Data from the Longitudinal Assessment of Bariatric Surgery-2 (LABS-2) study suggest an increase in AUD symptoms from 7% before surgery to 16% seven years after. Also, there is a higher representation of individuals who have had bariatric surgery in alcohol abuse treatment centres. Several risk factors appear to play a role in post-surgery alcohol problems. These factors include regular alcohol use before surgery, gender, age, and the type of surgery. 

While men tend to have more alcohol use and AUD than women in both the general population and among those who have had bariatric surgery, it is worth noting that most people seeking inpatient treatment for AUD after bariatric surgery are women. Research indicates that patients who undergo RYGB and SG, the most common bariatric surgeries, face a greater chance of dealing with alcohol issues after surgery compared to other surgeries or their condition before surgery. RYGB, in particular, has been linked to a higher chance of new AUD symptoms within five years after the procedure [24]. Higher risks of acquiring de novo alcohol-related illnesses, including alcoholic hepatitis, abuse, dependence, and poisoning, were independently linked to RYGB [25]. 

Although there is a lack of empirical evidence regarding factors that indicate problematic alcohol use following bariatric surgery, factors such as maleness, younger age, smoking, frequent alcohol use, pre-surgical alcohol use disorder, and a diminished sense of belonging have been found to predict alcohol misuse after the procedure. It is clear that it affects the possible mechanisms, such as particular bariatric surgery techniques, peptides and reinforcement/reward pathways, pharmacokinetics, and genetic factors [26]. 

Some may switch to lower-calorie foods, alcoholic drinks, or non-carbonated options, such as hard beverages, which could raise intoxication because of post-surgery metabolic changes. This drinking habit is worrisome, especially given the increased blood alcohol concentration relative to alcohol intake seen in bariatric surgery patients. The study checked RYGB's link to AUD versus LAGB, focusing on alcohol use and AUD at different times, broken down by surgery type. The results indicate that RYGB or LAGB patients upped their drinking frequency in the second year after surgery, compared to the year before or the first year after [19]. 

RYGB patients drank less on average during the first year post-surgery. Yet this was like pre-surgery numbers in the second. The rate of AUD rose during the second year compared to the first. LAGB patients saw no changes in drinking habits or AUD rates. 

In the review by Gregorio et al. [27], the aim is to focus on the high-risk criteria contraindicating the procedure, following American Society for Metabolic and Bariatric Surgery guidelines [28]. These involve past substance misuse, daily drinking before surgery, having Roux-en-Y gastric bypass, and smoking. Getting the true rate of alcohol abuse after surgery has been a point of interest. Studies show varying rates, with around 3.0% of surgery individuals possibly having alcohol-linked abuse [29,30]. Other studies mention post-surgery alcohol consumption rates ranging from 4.9% to 6.65%. 

Drinking habits after MBS can shift over time. Research finds alcohol use tends to rise by about 2% yearly post-surgery [26,31]. The timing of drinking use varies as well. In the beginning, six months after surgery, people usually start with alcohol at a lower rate, though, after a year, some people start to drink more. This could be because doctors advise against consuming alcohol in the first six months post-surgery. Some studies show that about 33% of cases include patients who drank more later on [19-21,32,33], while around 13% drank less. Long-term research points out that drinking goes down by about 9.1% after MBS [21]. 

Bramming et al. (2021) [12] did a study in Denmark from 2005 to 2013, looking at 14,309 people who had bariatric surgery and a control team of 53,279 who did not have surgery. To keep things fair, participants were chosen between 18 and 63 years old with a BMI of 32-60 kg/m². After leaving out studies, they ended up with 13,430 bariatric surgery patients and 21,021 non-surgical individuals. Through propensity score cutting and weighting, the final counts consisted of 12,251 in the surgery group and 16,005 in the non-surgery group. Further matching made 7,059 patients in each group [12]. Baseline stats initially differed but were evened out after statistical adjustments. Post-bariatric hypoglycemia (PBH) is an increasingly recognized complication after metabolic bariatric surgery and alcohol intake in RYGB is also associated with increased PBH due to insulin hyper-secretion that’s why this group is more vulnerable to this complication [34]. The research determined that people who chose to have bariatric surgery, mainly gastric bypass, were more likely to develop AUD than those who didn't. The risk remained for a long time, with the greatest risk showing up more than five years later. After about 6.9 years, those who had bariatric surgery had a 6-7 times higher risk of AUD compared to controls.

## Conclusions

This review shows there is a big risk of alcohol problems after MBS, especially if you had SG or RYGB. This means that it is super important to check people out carefully before surgery and keep an eye on how much they're drinking afterward. We need to understand how changes in your stomach, not getting enough nutrients, and alcohol problems all work together. This will help us take better care of people after surgery and cut down on bad outcomes. More studies are needed to figure out why people get more sensitive to alcohol and have more alcohol-related issues after these operations. If we know more, we can create better ways to help people with alcohol issues who are thinking about or have had MBS. 
